# Hospital toilets and drainage systems as a reservoir for a long-term polyclonal outbreak of clinical infections with multidrug-resistant *Klebsiella oxytoca* species complex

**DOI:** 10.1016/j.infpip.2024.100430

**Published:** 2024-12-21

**Authors:** Astri Lervik Larsen, Torunn Pedersen, Arnfinn Sundsfjord, Theodor A. Ross, Anja Dyresen Guleng, Jon Birger Haug, Anna K. Pöntinen, Ørjan Samuelsen

**Affiliations:** a Department of Infection Control, Østfold Hospital Trust, Sarpsborg, Norway; b Norwegian National Advisory Unit on Detection of Antimicrobial Resistance, Department of Microbiology and Infection Control, University Hospital of North Norway, Tromsø, Norway; c Department of Medical Biology, Faculty of Health Sciences, UiT The Arctic University of Norway, Tromsø, Norway; d Department of Physics and Technology, UiT The Arctic University of Norway, Tromsø, Norway; e Centre for Laboratory Medicine, Østfold Hospital Trust, Sarpsborg, Norway; f Department of Biostatistics, Faculty of Medicine, University of Oslo, Oslo, Norway

**Keywords:** *Klebsiella* species, Hospital, Toilets, Drainage systems, Multidrug-resistant micro-organisms, Hospital-acquired infection

## Abstract

**Background:**

Nosocomial outbreaks with multidrug-resistant bacteria with a probable reservoir in hospital toilets and drainage systems have been increasingly reported.

**Aim:**

To investigate an increase in bacteraemia with extended-spectrum β-lactamase (ESBL)-producing *Klebsiella oxytoca* at our hospital in 2021; the epidemiology of the outbreak suggested an environmental source.

**Methods:**

Available clinical *K. oxytoca* isolates from patient with infection or rectal carriage from 2019 to 2022 were collected. Clinical information was gathered from included patients and sampled sinks, shower drains, and toilet water. Short- and long-read whole-genome sequencing (WGS) was performed on patient and environmental isolates to assess phylogenetic relationships, antibiotic resistance genes/mutations, and plasmid profiles.

**Results:**

WGS revealed four clusters and a polyclonal population consisting of ESBL-producing *K. oxytoca* and *Klebsiella michiganensis*. All clusters contained both clinical and environmental isolates. The environmental sampling revealed widespread contamination of the outbreak strains in the outbreak ward, and plasmid analyses indicated possible transfer of plasmids between species and clones. Most environmental findings in the outbreak ward were from toilet water, and enhanced cleaning of bathrooms and toilets was introduced. The following year, a decrease in outbreak strains in systemic infections was observed.

**Conclusion:**

This investigation uncovered a polyclonal outbreak of multidrug-resistant *K. oxytoca* and *K. michiganensis* and unveiled a persistent reservoir of outbreak clones in the drainage system and toilet water, facilitating exchange of resistance genes. The risk of toilet water as a source of clinical infections warrants further investigation.

## Introduction

Adherence to standard precautions for infection control is paramount in controlling hospital outbreaks of multidrug-resistant (MDR) bacteria. Careful hand hygiene is particularly important along with surveillance cultures and contact precautions for colonized patients [[1], [2], [3]]. However, should new cases appear despite these measures, it is essential to investigate alternative factors supporting continued spread of infection, including persistent environmental reservoirs.

Recent studies have highlighted the importance of bacterial biofilms within hospital drainage systems as reservoirs for hospital infections. Drainage system-associated biofilms may house MDR organisms and other nosocomial pathogens and might support prolonged outbreaks [[4], [5], [6], [7], [8]]. A key challenge arises from the persistence and heterogeneity of species and strains within environmental reservoirs, which complicates outbreak detection and creates conditions supporting horizontal gene transfer encoding antimicrobial resistance [6,9,10].

*K. oxytoca* is commonly found in the human intestinal tract. Taxonomic studies have shown that *K. oxytoca* is a member of the *K. oxytoca* species complex (KoSC), which also includes *Klebsiella michiganensis*, *Klebsiella grimontii*, *Klebsiella huaxiensis*, *Klebsiella pasteurii*, and *Klebsiella spallanzanii* [11]. Conventional methods of identification (e.g. matrix-assisted laser desorption/ionization time-of-flight mass spectrometry (MALDI-TOF)) are often unable to identify the members of this complex at the species level. Although typically regarded as an opportunistic pathogen, there is increasing evidence of *K. oxytoca* causing nosocomial infections and outbreaks, and environmental sources include wastewater drainage systems, handwashing sinks, and contaminated detergent [[11], [12], [13], [14], [15]].

At Østfold Hospital, an unexpected rise in bacteraemia with extended-spectrum β-lactamase (ESBL; CTX-M)-producing *K. oxytoca* was identified in the late summer of 2021. In the outbreak investigation we collected retrospective and prospective data on cases with ESBL-producing *K. oxytoca*, conducted environmental screening, and performed genomic analysis of selected isolates. We hypothesized that a persistent environmental reservoir contributed to the increase in ESBL-producing *K. oxytoca*.

## Methods

### Study design

First, a retrospective outbreak investigation was conducted to identify risk factors for the acquisition of ESBL-producing *K. oxytoca*. In all, 21 patients were included: those with a bloodstream infection in 2021 (*N* = 7), an accessible clinical isolate (bloodstream infection or urinary tract infection) from 2019 to 2022 (*N* = 12), and a rectal carriage (*N* = 2) identified in another outbreak investigation. The corresponding hospital records were examined and the following information was collected: (i) prior admission to the Østfold Hospital within the past three months, and the ward(s) involved, (ii) prior admission to other hospitals in Norway or abroad within the past three months, and (iii) endoscopy procedures performed during the past three months. Second, aqueous environmental samples from patient rooms were collected from the ward that – based on the results from the retrospective investigations – was the likely outbreak ward, and to a lesser extent from other clinical wards. Finally, all bacterial isolates from the included patients and a selection of isolates from the environment (*N* = 9) were subjected to whole-genome sequencing (WGS). The retrospective investigation was performed towards the end of 2021, and enhanced cleaning interventions were implemented at the start of 2022. Environmental sampling was conducted during three periods of 2022.

### Environmental screening

Toilet water samples were collected using a 20 mL syringe (Omnifix Luer Solo; B. Braun, Melsungen, Germany), transferred to sterile containers (Universalcontainer PS; Deltalab, Barcelona, Spain), and centrifuged precipitates were cultured on selective media (see below). Shower drain samples were collected targeting below the water trap, because of the proximity to the drainage pipes. In the outbreak ward, sink drainers and faucet in all the patient rooms were sampled. Shower drain, sink drain, and faucet samples were collected using sterile swabs (Eswab; Copan, Brescia, Italy).

### Microbiological analysis

Screening samples were cultured on ESBL-selective media (CHROMagar ESBL; CHROMagar, Paris, France). MALDI-TOF (Bruker Daltonik, Bremen, Germany) was used for species identification. Antimicrobial susceptibility testing was performed using disc diffusion (Oxoid, Basingstoke, UK) according to EUCAST [16]. Phenotypic ESBL production was confirmed with clavulanate synergy using double disc synergy test (Oxoid) or combination disk test (Rosco Neo-Sensitabs, Taastrup, Denmark). Extended susceptibility testing of a selection of isolates was performed by broth microdilution using Sensititre microtitre plates (Trek Diagnostic Systems/ThermoFisher Scientific, East Grinstead, UK). Interpretation of antimicrobial susceptibility was according to EUCAST guidelines [17].

### Genomic analyses

Genomic DNA was extracted using the EZ1 platform (Qiagen, Hilden, Germany). For Oxford Nanopore Technologies (ONT; Oxford, UK) sequencing, DNA was further purified by AMPure XP beads (A63882; Beckman Coulter, Krefeld, Germany), and libraries were generated using the Rapid Barcoding kit (SQK-RBK004). Sequencing and base calling (fast mode) were performed on MinION Mk1C using FLO-MIN106 flow cells. For Illumina sequencing, 2×151 bp paired-end libraries were prepared using Nextera®XT, and sequenced on the NextSeq550 platform (Illumina, San Diego, CA, USA). Quality control of Illumina sequence and assembly data was performed using FastQC v.0.11.9 and Quast v.5.2.0 (thresholds: minimum 40× coverage; maximum 400 contigs). Species definition was confirmed using rmlst [18]. For Illumina reads, assembly was performed using Shovill v.1.1.0 (https://github.com/tseemann/shovill), and for Illumina–ONT hybrid using Unicycler v.0.4.8 in normal mode [19]. Bakta v.1.7.0 was used for annotation [20]. Sequence types (STs) were retrieved using mlst v.2.23.0 (https://github.com/tseemann/mlst) and the *K. oxytoca* database (https://pubmlst.org/) [21]. Pangenome for *K. oxytoca* were estimated using Panaroo v.1.3.2 with the sensitive mode, merging paralogs and removing invalid genes, and core genes defined using a 99% threshold for presence [22]. Maximum-likelihood phylogenies for *K. oxytoca* were separately inferred from their core genomes using RAxML v.8.2.12 with the GTR + Gamma rate model and 100 bootstraps [23]. Antimicrobial resistance genes/point mutations were identified using AMRFinderPlus v.3.11.2, database version v.2023-08-08.2 with minimum identity and coverage of 90%. Plasmid replicons were retrieved using PlasmidFinder v.2.1.6. For cluster-specific analyses, *K. oxytoca* ST323 and ST223 were separately mapped to the *K. oxytoca* reference genome ASM381292v1 (GCF_003812925.1) and *K. michiganensis* ST66 and ST376 to the *K. michiganensis* genome assembly ASM917348v1 (GCF_009173485.1). Pairwise single-nucleotide polymorphism (SNP) distances were calculated from alignments using the Nullarbor pipeline v.2.0.20191013 (https://github.com/tseemann/nullarbor). Visualizations were produced in R v.4.3.1. or EasyFig [24,25].

### Statistical analysis

The proportion of ESBL-producing *K. oxytoca* was modelled by a logistic regression using location (local and national) and time (2016–2022) as covariates. An interaction term between time and location allowed for different progress over the years for the two locations. The model furthermore accounted for the intervention in 2022 at the local hospital. The national data was acquired from the Norwegian Surveillance System on Resistant Microbes (NORM) and included the local (Østfold Hospital) data within the yearly nine-month national data collection periods [26].

## Results

### Increasing proportion of ESBL-producing *K. oxytoca*

**Figure 1:**
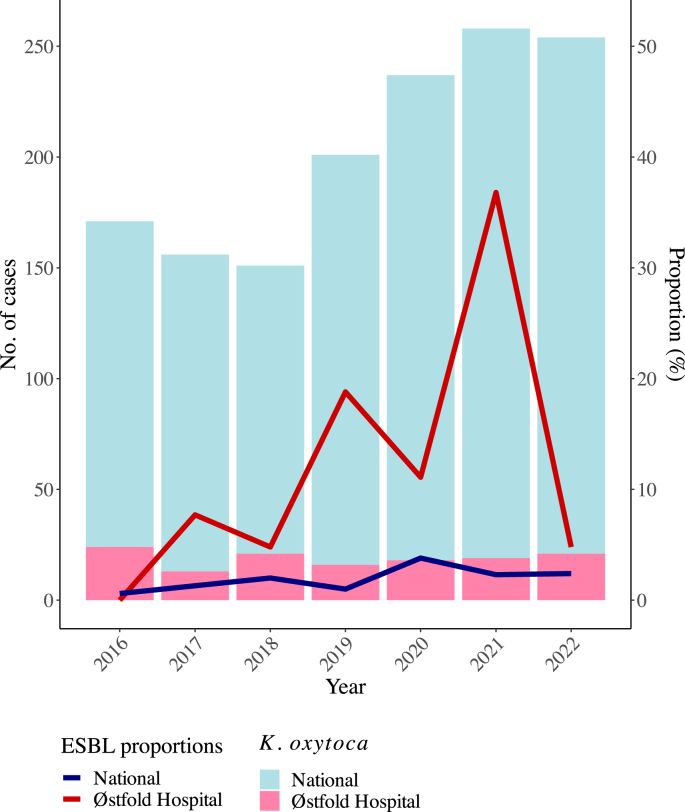
Number of cases of *Klebsiella oxytoca* (stacked bars) and proportion of extended-spectrum β-lactamase (ESBL)-producing *K. oxytoca* (lines) among all detected blood culture isolates of *K. oxytoca* at Østfold Hospital compared to the national level during the years 2016–2022 [26].

During 2021, an unusual increase in the proportion of bacteraemia cases with ESBL-producing *K. oxytoca* was observed at the Østfold Hospital in Norway (Figure 1), from 0% in 2016 to 37% in 2021. The overall number of *K. oxytoca* from blood culture remained relatively unchanged during the same period with an average of 19 cases per/year (range: 13–24). In contrast, the national proportion of ESBL among *K. oxytoca* blood culture isolates was lower and comparatively stable (0.6% in 2016 to 2.3% in 2021), indicating a local rather than a national trend. The logistic regression showed significant (*P* < 0.05) differences in the proportions of ESBL-producing *K. oxytoca* between the two sites in years 2019–2021.

Analyses of the 21 included cases with ESBL-producing *K. oxytoca* from 2019 to 2021 (Table I) showed that all had been admitted to our hospital within three months before detection. Over the preceding year, most patients also had multiple admissions across different wards. The ward with the most case-linked admissions within three months before ESBL-producing *K. oxytoca* bacteraemia was the surgical ward of urology, vascular surgery, and otorhinolaryngology (UVO ward). None of the patients had been hospitalized abroad in the 12 months preceding the identification of ESBL-producing *K. oxytoca*. Five patients had been recently admitted to another Norwegian hospital, but only two patients had stayed at the same hospital. In the three-month period before detection, five, two, and one of the included patients had undergone cystoscopy, gastroscopy, or colonoscopy, respectively. One patient had undergone endoscopic retrograde cholangiopancreatography, but at another hospital.

**Table I:** Relevant epidemiological and genomic characteristics of included isolates

Isolate ID	Sample material	Sample date (month/year)	*Klebsiella* species	ST	*Bla*_CTX-M_ variant	*Bla*_CTX-M_ location
Cluster 1						
kreshist0054	Blood	03/2019	*K. michiganensis*	66	*bla*_CTX-M-15_	Plasmid, chromosome
kreshist0055	Blood	10/2019	*K. michiganensis*	66	*bla*_CTX-M-15_	ND
kreshist0056	Toilet room A	02/2022	*K. michiganensis*	66	*bla*_CTX-M-15_	ND
kreshist0057	Toilet water room B	06/2022	*K. michiganensis*	66	*bla*_CTX-M-15_	ND
kreshist0058	Shower drain room C	06/2022	*K. michiganensis*	66	*bla*_CTX-M-15_	ND
kreshist0059	Toilet water room D	06/2022	*K. michiganensis*	66	*bla*_CTX-M-15_	ND
Cluster 2						
kreshist0063	Blood	12/2019	*K. oxytoca*	223	*bla*_CTX-M-15_	ND
kreshist0062	Blood	02/2021	*K. oxytoca*	223	*bla*_CTX-M-15_	Plasmid
kreshist0064	Toilet corridor	02/2022	*K. oxytoca*	223	*bla*_CTX-M-15_	ND
Cluster 3						
kreshist0068	Blood	01/2021	*K. oxytoca*	323	*bla*_CTX-M-154_	Plasmid, chromosome
kreshist0066	Urine	08/2021	*K. oxytoca*	323	*bla*_CTX-M-154_	ND
kreshist0065	Feces	08/2021	*K. oxytoca*	323	*bla*_CTX-M-154_	ND
kreshist0067	Urine	08/2021	*K. oxytoca*	323	*bla*_CTX-M-154_	ND
kreshist0069	Urine	11/2021	*K. oxytoca*	323	*bla*_CTX-M-154_	ND
kreshist0070	Urine	02/2022	*K. oxytoca*	323	*bla*_CTX-M-154_	ND
kreshist0071	Toilet room E	02/2022	*K. oxytoca*	323	*bla*_CTX-M-154_	ND
kreshist0072	Toilet water room F	06/2022	*K. oxytoca*	323	*bla*_CTX-M-154_	ND
Cluster 4						
kreshist0073	Blood	01/2021	*K. michiganensis*	376	*bla*_CTX-M-15_	Chromosome
kreshist0076	Blood	08/2021	*K. michiganensis*	376	*bla*_CTX-M-15_	ND
kreshist0074	Urine	09/2021	*K. michiganensis*	376	*bla*_CTX-M-15_	ND
kreshist0075	Urine	10/2021	*K. michiganensis*	376	*bla*_CTX-M-15_	ND
kreshist0077	Blood	11/2021	*K. michiganensis*	376	*bla*_CTX-M-15_	ND
kreshist0078	Feces	12/2021	*K. michiganensis*	376	*bla*_CTX-M-15_	ND
kreshist0079	Toilet room A	02/2022	*K. michiganensis*	376	*bla*_CTX-M-15_	ND
kreshist0080	Shower drain room E	06/2022	*K. michiganensis*	376	*bla*_CTX-M-15_	ND
Non-clustered isolates						
kreshist0053	Blood	02/2020	*K. michiganensis*	52	*bla*_CTX-M-15_	Plasmid
kreshist0081	Blood	07/2021	*K. michiganensis*	384	*bla*_CTX-M-15_	Plasmid
kreshist0060	Urine	10/2021	*K. michiganensis*	183	*bla*_CTX-M-154_	Chromosome
kreshist0061	Blood	11/2021	*K. oxytoca*	199	*bla*_CTX-M-15_	Plasmid
kreshist0052	Blood	03/2022	*K. michiganensis*	11	*bla*_CTX-M-15_	ND

ST, sequence type; ND, not determined.

### Environmental screening

The strong association with admissions to a specific ward and previous *K. oxytoca* outbreak characteristics indicated a potential environmental reservoir. Consequently, during three periods in 2022 (February, June, and August), 251 aqueous environmental samples were collected from the affected ward and from adjacent and distantly located wards for comparison. Contact points were also sampled in designated rooms. The environmental screening unveiled growth of ESBL-producing *K. oxytoca* in multiple wards in a total of 36/235 (15.3%) samples (Table II).

**Table II:** Findings of ESBL-producing *Klebsiella oxytoca* in the drainage system of different hospital wards including all aqueous environmental samples collected during 2022

Ward	ESBL-producing *K. oxytoca* positive rooms/rooms sampled (%)
Gastro surgery	4/27 (14.8%)
Urology, vascular surgery, otorhinolaryngology	15/26 (57.7%)
Nephrology, geriatrics, gastrointestinal diseases	1/9 (11.1%)
Oncology	1/26 (3.8%)
Respiratory diseases	0/9 (0%)
Children's ward	1/6 (16.7%)
Maternity ward	0/8 (0%)
Infectious diseases	1/24 (4.2%)

ESBL, extended-spectrum β-lactamase.

The UVO ward was predominantly affected, with ESBL-producing *K. oxytoca* detected in 58% (15/26) of the rooms. No environmental contamination was observed in the respiratory disease and maternity wards, though the sample number was small. In the UVO ward, defined as the outbreak ward, toilet bowl water reservoirs emerged as the predominant environmental source with 81% (21/26) of samples supporting growth of ESBL-producing *K. oxytoca*, in contrast to 19% (5/26) and 8% (2/26) of the shower and sink drain samples, respectively.

### Interventions

Based on the results from the environmental samples, enhanced cleaning of the bathrooms was implemented in the most affected hospital wards. Disinfection was performed twice a day during most of 2022, including disinfecting the toilet water trap. Different disinfectants were applied initially, finally selecting disinfection of toilets, shower drains and sinks with Perasafe (Brage Medical AS, Drammen, Norway) and deposition of 50 mg (10 tablets) Rely+On Virkon (Lanxess, Köln, Germany) in the toilet water trap at the end of cleaning as the routine. The introduction of targeted measures specifically addressing the cleaning and disinfection of toilets and bathrooms had an abrupt effect on the occurrence of ESBL-producing *K. oxytoca* blood culture isolates (Figure 1).

### Genomic and microbiological characterization

To explore the increased incidence of ESBL-producing *K. oxytoca*, short-read WGS was performed for all clinical isolates and a subset of environmental isolates (*N* = 9), 30 in total (Table I and Supplementary Table S1).

**Figure 2:**
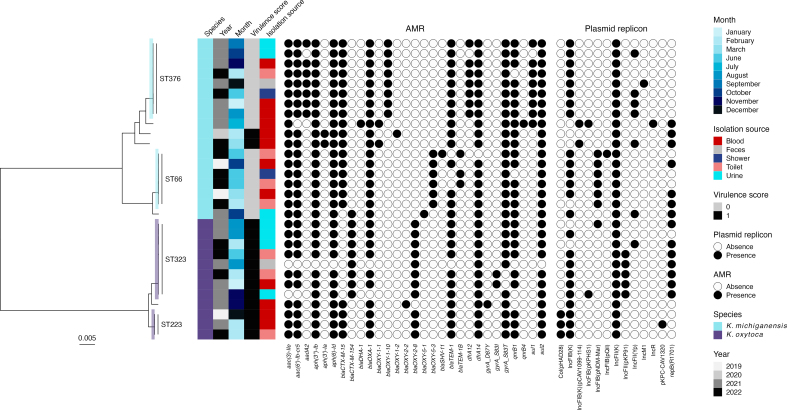
Maximum-likelihood phylogeny on core genome alignment, aligned with species information, temporal data, virulence scores, isolation sources, resistance profiles, and plasmid replicons of the environmental and clinical *Klebsiella michiganensis* and *Klebsiella oxytoca* isolates selected for whole-genome sequencing.

The sequencing revealed two species, *K. oxytoca* and *K. michiganensis*, each displaying two clusters: *K. oxytoca* ST323 and ST223, and *K. michiganensis* ST376 and ST66 (Table I, Figure 2). All clusters contained both clinical and environmental isolates, confirming the overlap between the human and environmental niche(s). The SNP distance within each cluster varied; ST323 (15–39 SNP), ST223 (11–64 SNP), ST376 (18–76 SNP), and ST66 (36–68 SNP). In addition, single isolates of *K. michiganensis* ST11, ST52, ST183, ST384, and *K. oxytoca* ST199 were identified, all associated with infections, but from patients who had not been admitted to the outbreak ward in the period before detection.

The isolates within both *K. michiganensis* clusters and *K. oxytoca* ST223 harboured *bla*_CTX-M-15_. In contrast, *K. oxytoca* ST323 carried *bla*_CTX-M-154_, a single nucleotide variant of *bla*_CTX-M-15_. Notably, *bla*_CTX-M-154_ was also identified in the *K. michiganensis* ST183 isolate, whereas *bla*_CTX-M-15_ was present in the other single ST isolates. This dissemination pattern suggests the genetic sharing of one common resistance determinant but with a single mutation in the CTX-M-encoding gene. Resistance profiles, including additional resistance genes and mutations, generally followed the cluster and ST profile, albeit with some variability (Figure 2 and Supplementary Table S1). Each ST featured a distinct variant of the intrinsic OXY β-lactamase. Except for one isolate, all were classified as MDR.

The plasmid replicon profile varied both between clusters and ST, as well as within clusters. Nonetheless, the consistent presence of the IncFII(K) replicon in all isolates (Figure 2) suggested a potential role of this plasmid type in the dissemination of the ESBL-encoding genes. To explore this hypothesis and investigate the ESBL determinant's genetic surroundings, additional long-read sequencing was conducted for eight isolates representing each cluster and the individual STs.

**Figure 3:**
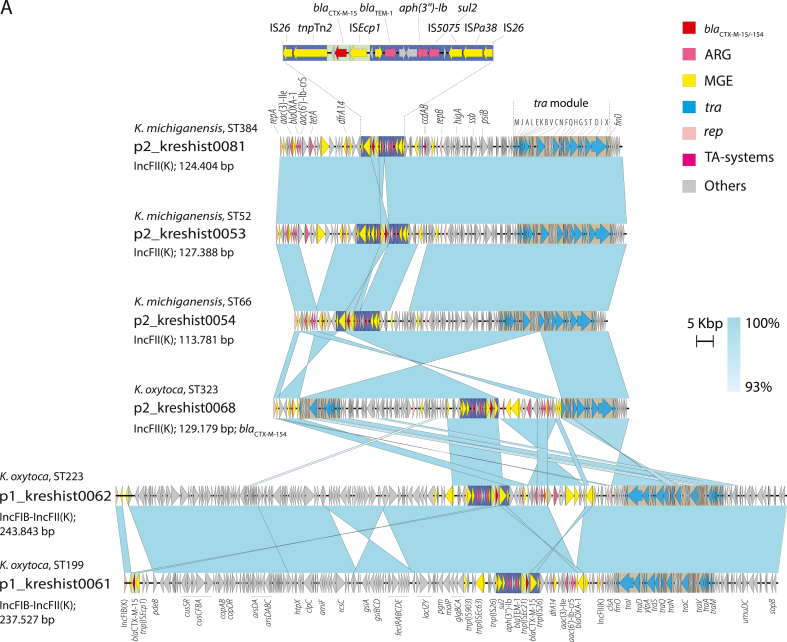

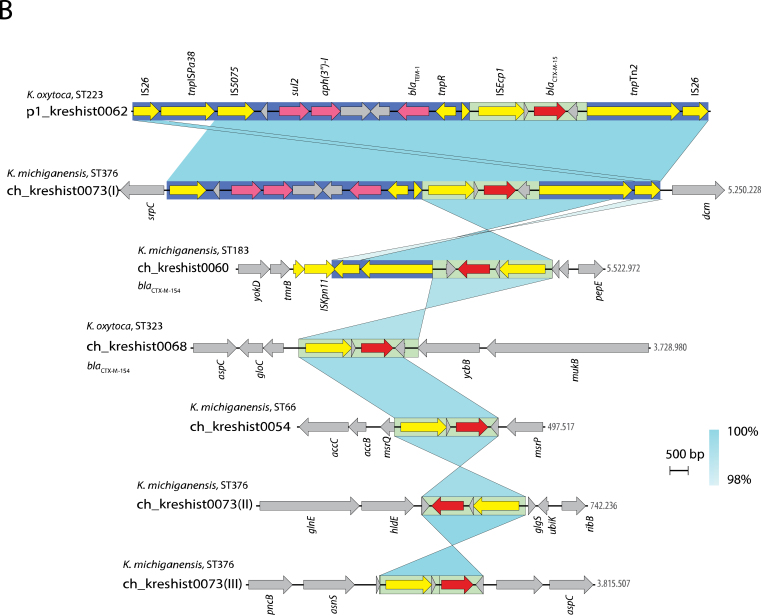
Genetic context of the extended-spectrum β-lactamase (ESBL)-encoding gene in *Klebsiella oxytoca* and *Klebsiella michiganensis* from the outbreak investigation. Comparisons of plasmids (p) in (A) and chromosomal (ch) regions (B) containing *bla*_CTX-M-15/-154_ for the indicated isolates. Turquoise shading between pairs of sequences indicates identity (93–100% or 98–100%). Annotated CDS representing *bla*_CTX-M-_ and other antibiotic resistance genes (ARG), mobile genetic elements (MGE), genes involved in conjugative transfer (*tra*), replication initiation (*rep*), toxin–antitoxin (TA) systems, and others are represented by arrows with the given colour codes. Regions included in the ∼16 kb (blue) and ∼3 kb (light green) *bla*_CTX-M_ elements and the transfer module (dark grey) are boxed. The specific positions are provided for the chromosomal integrations.

Sequence comparisons revealed that six isolates contained IncFII(K) plasmids carrying *bla*_CTX-M-15/-154_ (Figure 3A). The plasmids identified in *K. michiganensis* (ST52, ST66, and ST384) and *K. oxytoca* (ST323) exhibited high sequence identity (99.7–99.9%) with coverage ranging from 94% to 100%, suggesting potential plasmid transmission. Despite minor observed inversions and insertions, these plasmids shared gene content and synteny, including a second replicon repB(R1701), resistance encoding genes, and a complete transfer module, supporting their capability for conjugative transfer. In two isolates, *K. oxytoca* ST199 and ST223, *bla*_CTX-M-15_ was located on similar IncFII(K)-IncFIB hybrid plasmids (∼240 kb), sharing the set of resistance genes and a putative transfer module, but otherwise distinct from the first plasmid group.

However, all six plasmids contained *bla*_CTX-M-15/-154_ along with additional resistance genes (*sul2*, *aph(3″)-Ib*, and *bla*_TEM-1_) and IS elements on a common ∼16 kb region (Figure 3B). This genetic element was bounded by direct repeats of IS*26*, as described for highly transferable class I transposons [27]. This element (100% coverage and 100% identity) appeared in diverse plasmids (*N* = 73) within the NCBI completed plasmid database of Klebsiella (taxid:570). None was closely related to those in our study, supporting its mobility. Among the plasmids in the current study, two variants of this *bla*_CTX-M_ element were observed: a short truncation in *K. oxytoca* ST323 and a duplication in *K. michiganensis* ST52. This duplicated region corresponds precisely to an ∼3 kb segment containing IS*Ecp1* and *bla*_CTX-M-15_, also found as a second *bla*_CTX-M-15_ copy in the *K. oxytoca* ST199 plasmid (Figure 3A) and as chromosomal insertions (Figure 3B).

In ST376 (*bla*_CTX-M-15_) and ST183 (*bla*_CTX-M-154_) the ESBL gene was located on the chromosome rather than within their IncFII(K) plasmids; for ST376 in three copies (I, II, and III). In contrast to the plasmid-encoded elements, these chromosomal insertions were delineated by IS*5075* or IS*Ecp1* together with IS*26* due to 5′-end truncations (Figure 3B). Additionally, we detected insertions of the ∼3 kb IS*Ecp1*-*bla*_CTX-M_ element into various chromosomal positions in *K. michiganensis* ST66 and *K. oxytoca* ST323, where the CTX-M-encoding element was also present within the plasmid. The finding of *bla*_CTX-M_ elements inserted into chromosomal positions and in different genetic backgrounds indicates independent transposition events.

These findings show that the IncFII(K) replicon type plasmid facilitates the horizontal spread of the ESBL determinant across distinct species and STs. Additionally, this study highlights IS-mediated mobilization of flexible-sized CTX-M-encoding modules between and within plasmids and chromosomes.

## Discussion

Contaminated toilet and water drainage systems have increasingly been recognized as reservoirs for nosocomial outbreaks with MDR bacteria [[4], [5], [6], [7], [8]]. In our outbreak investigation, we hypothesized that biofilm formation within the hospital drainage system created a diverse and persistent reservoir of bacteria that could infect patients. Phylogenetic analysis identified genetically and timely related isolates from clinical and environmental samples, supporting our hypothesis of a shared origin. The detection of highly conserved genetic elements responsible for the dissemination of the ESBL phenotype between species and strains, along with the discovery of outbreak clusters dated back to 2019, strongly indicates the presence of a persistent, mixed outbreak originating from a common reservoir.

The identified high-risk hospital ward also had the highest prevalence of positive environmental samples, including ESBL-producing *K. oxytoca* in 21 out of 26 toilets. Transmission of bacteria from toilet water to patients may occur through aerosolization during flushing and surface contamination of contact points [[28], [29], [30], [31]]. Toilet flushes can produce a strong, chaotic jet capable of transporting aerosols, which may contain micro-organisms from faecal waste [30,32]. Indeed, toilets have been identified as a source of hospital contamination and associated outbreaks [[33], [34], [35]].

In an outbreak of OXA-48-producing *K. pneumoniae*, where toilet drain water was suspected to be the source of room-to-room transmission, several drainpipe obstructions occurred leading to the retrograde flow of wastewater, and disinfection efforts were only temporarily successful [33]. The likelihood of room-to-room transmission via the drainage system is diminished in our hospital due to few horizontal drainpipes. However, factors such as high patient turnover, frequent readmissions and switching of rooms may have contributed to the spread of the outbreak strains. Nevertheless, the enhanced disinfection, targeting toilets, shower drains, and sinks led to a notable reduction in the number of bloodstream infections caused by ESBL-producing *K. oxytoca/michiganensis* in 2022 compared to 2021. This outcome further emphasizes the importance of the environment as the source for transmission.

Only a small number of studies on hospital outbreaks due to *K. oxytoca* have determined the specific species/STs involved (as reviewed by [11]), leaving the differential outbreak potential among members of the *K. oxytoca* species complex unclear. Our study identified two species within the *K. oxytoca* complex (*K. oxytoca* and *K. michiganensis*) implicated in the outbreak. It underscores the importance of accurate species identification and demonstrates that other species within the *K. oxytoca* species complex may contribute to outbreaks. Among the STs identified, *K. oxytoca* ST223 and ST323 have been identified from clinical samples in multiple countries, including the USA, Australia, Switzerland, and Denmark, indicating a global distribution of these lineages [36,37].

Long-read sequencing enabled a comprehensive characterization of the genomic architecture of the ESBL-encoding elements shared among the outbreak bacteria, providing valuable insights into transmission mechanisms. Comparative analyses of the *bla*_CTX-M_-containing IncFII(K) plasmids revealed several cases of horizontal transfer within and among the two *Klebsiella* species, consistent with observations in previous outbreaks [6,9,10]. The location of *bla*_CTX-M_ on both plasmids and chromosomes illustrates the dynamic nature of resistance genes linked to IS elements, where involvement of IS*26* is known to mediate efficient transposition [27]. These findings underscore the complexity of outbreaks originating from environmental sources challenging outbreak investigations.

Our study has several limitations. The selection of a subset of isolates for genomic investigation posed a limitation in fully elucidating the extent of the outbreak and obtaining a comprehensive understanding of it. Moreover, we did not systematically screen patients in the affected wards, and the environmental screening was not initiated before 2022. Consequently, we lack data on asymptomatic colonization and cannot determine when the strains were initially established in the environment. Screening patients in and out of the hospital ward could have strengthened the assumption that patients are infected during admission. However, the identification of genetically closely related clinical isolates more than three years before the environmental screening underlines long-term environmental contamination.

## Conclusion

This study has investigated a polyclonal hospital outbreak of MDR *K. oxytoca* species complex causing urinary tract infections and invasive disease. It delineates the dissemination of the outbreak bacteria in the wastewater system of the implicated hospital wards, investigating the relatedness between isolates from patients and the environment, along with the genetic context of the ESBL determinant. Our findings underscore the persistence of resistant micro-organisms within sewage pipes, the capability of the bacteria to migrate from the pipes to toilet bowls, and their role as an environmental source of serious nosocomial infections. Consequently, toilets should be recognized as a reservoir for nosocomial transmission of MDR bacteria, affecting hospital hygiene protocols and cleaning procedures.

## CRediT author statement

**Astri Lervik Larsen:** Conceptualization, Methodology, Resources, Writing - Original Draft, Investigation.

**Torunn Pedersen:** Methodology, Validation, Formal analysis, Resources, Data Curation, Visualization.

**Arnfinn Sundsfjord:** Writing - Review & Editing.

**Theodor A. Ross:** Visualization.

**Anja Dyresen Guleng:** Validation, Writing - Original Draft.

**Jon Birger Haug:** Writing - Original Draft.

**Anna K. Pöntinen:** Methodology, Visualization, Formal analysis, Resources, Data Curation.

**Ørjan Samuelsen:** Supervision, Writing - Original Draft, Writing - Review & Editing, Resources, Validation.

## Ethics approval

Ethical approval was not required after evaluation by the Regional Ethical Committee (REK South-East A, 553976). The project was approved and defined as a quality assurance project by the Data Protection Officer at Østfold Hospital Trust (22/07500-3).

## Data availability

Whole-genome sequences are available in the European Nucleotide Archive under BioProject PRJEB76256.

## Funding statement

Part of this work was supported by a grant from the Norwegian Society of Medical Microbiology.

## Conflict of interest statement

None declared.

## References

[1] Pryor R., Viola-Luqa C., Hess O., Bearman G. (2020). Barrier precautions in the era of multidrug pathogens. Curr Treat Options Infect Dis.

[2] Campos A.C., Albiero J., Ecker A.B., Kuroda C.M., Meirelles L.E., Polato A. (2016). Outbreak of *Klebsiella pneumoniae* carbapenemase-producing *K pneumoniae*: a systematic review. Am J Infect Control.

[3] Mills J.P., Marchaim D. (2021). Multidrug-resistant Gram-negative bacteria: infection prevention and control update. Infect Dis Clin North Am.

[4] Hamerlinck H., Aerssens A., Boelens J., Dehaene A., McMahon M., Messiaen A.S. (2023). Sanitary installations and wastewater plumbing as reservoir for the long-term circulation and transmission of carbapenemase producing *Citrobacter freundii* clones in a hospital setting. Antimicrob Resist Infect Control.

[5] Vergara-López S., Domínguez M.C., Conejo M.C., Pascual Á., Rodríguez-Baño J. (2013). Wastewater drainage system as an occult reservoir in a protracted clonal outbreak due to metallo-β-lactamase-producing *Klebsiella oxytoca*. Clin Microbiol Infect.

[6] Valzano F., Coda A.R.D., Liso A., Arena F. (2024). Multidrug-resistant bacteria contaminating plumbing components and sanitary installations of hospital restrooms. Microorganisms.

[7] Choquet M., Mullié C. (2022). Down the drain: a systematic review of molecular biology evidence linking sinks with bacterial healthcare-associated infections in intensive care units. Hygiene.

[8] Kizny Gordon A.E., Mathers A.J., Cheong E.Y.L., Gottlieb T., Kotay S., Walker A.S. (2017). The hospital water environment as a reservoir for carbapenem-resistant organisms causing hospital-acquired infections – a systematic review of the literature. Clin Infect Dis.

[9] Tofteland S., Naseer U., Lislevand J.H., Sundsfjord A., Samuelsen Ø. (2013). A long-term low-frequency hospital outbreak of KPC-producing *Klebsiella pneumoniae* involving Intergenus plasmid diffusion and a persisting environmental reservoir. PLoS One.

[10] Ory J., Bricheux G., Robin F., Togola A., Forestier C., Traore O. (2019). Biofilms in hospital effluents as a potential crossroads for carbapenemase-encoding strains. Sci Total Environ.

[11] Yang J., Long H., Hu Y., Feng Y., McNally A., Zong Z. (2022). *Klebsiella oxytoca* complex: update on taxonomy, antimicrobial resistance, and virulence. Clin Microbiol Rev.

[12] Neog N., Phukan U., Puzari M., Sharma M., Chetia P. (2021). *Klebsiella oxytoca* and emerging nosocomial infections. Curr Microbiol.

[13] Leitner E., Zarfel G., Luxner J., Herzog K., Pekard-Amenitsch S., Hoenigl M. (2015). Contaminated handwashing sinks as the source of a clonal outbreak of KPC-2-producing *Klebsiella oxytoca* on a hematology ward. Antimicrob Agents Chemother.

[14] Lowe C., Willey B., O’Shaughnessy A., Lee W., Lum M., Pike K. (2012). Outbreak of extended-spectrum β-lactamase-producing *Klebsiella oxytoca* infections associated with contaminated handwashing sinks. Emerg Infect Dis.

[15] Chapman P., Forde B.M., Roberts L.W., Bergh H., Vesey D., Jennison A.V. (2020). Genomic investigation reveals contaminated detergent as the source of an extended-spectrum-β-lactamase-producing *Klebsiella michiganensis* outbreak in a neonatal unit. J Clin Microbiol.

[16] Matuschek E., Brown D.F., Kahlmeter G. (2014). Development of the EUCAST disk diffusion antimicrobial susceptibility testing method and its implementation in routine microbiology laboratories. Clin Microbiol Infect.

[17] European Committee on Antimicrobial Susceptibility Testing (2024). Breakpoint tables for interpretation of MICs and zone diameters 2024; Version 14.0. http://www.eucast.org.

[18] Jolley K.A., Bliss C.M., Bennett J.S., Bratcher H.B., Brehony C., Colles F.M. (2012). Ribosomal multilocus sequence typing: universal characterization of bacteria from domain to strain. Microbiol.

[19] Wick R.R., Judd L.M., Gorrie C.L., Holt K.E. (2017). Unicycler: resolving bacterial genome assemblies from short and long sequencing reads. PLoS Comput Biol.

[20] Schwengers O., Jelonek L., Dieckmann M.A., Beyvers S., Blom J., Goesmann A. (2021). Bakta: rapid and standardized annotation of bacterial genomes via alignment-free sequence identification. Microb Genom.

[21] Jolley K.A., Bray J.E., Maiden M.C.J. (2018). Open-access bacterial population genomics: BIGSdb software, the PubMLST.org website and their applications. Wellcome Open Res.

[22] Tonkin-Hill G., MacAlasdair N., Ruis C., Weimann A., Horesh G., Lees J.A. (2020). Producing polished prokaryotic pangenomes with the Panaroo pipeline. Genome Biol.

[23] Stamatakis A. (2014). RAxML version 8: a tool for phylogenetic analysis and post-analysis of large phylogenies. Bioinformatics.

[24] R Core Team (2020). https://www.r-project.org/.

[25] Sullivan M.J., Petty N.K., Beatson S.A. (2011). Easyfig: a genome comparison visualizer. Bioinformatics.

[26] NORM/NORM-VET. NORM/NORM-VET 2022 (2023).

[27] Harmer C.J., Hall R.M. (2016). IS*26*-mediated formation of transposons carrying antibiotic resistance genes. mSphere.

[28] Wilson G.M., Jackson V.B., Boyken L.D., Schweizer M.L., Diekema D.J., Petersen C.A. (2020). Bioaerosols generated from toilet flushing in rooms of patients with *Clostridioides difficile* infection. Infect Control Hosp Epidemiol.

[29] Knowlton S.D., Boles C.L., Perencevich E.N., Diekema D.J., Nonnenmann M.W. (2018). Bioaerosol concentrations generated from toilet flushing in a hospital-based patient care setting. Antimicrob Resist Infect Control.

[30] Best E.L., Sandoe J.A., Wilcox M.H. (2012). Potential for aerosolization of *Clostridium difficile* after flushing toilets: the role of toilet lids in reducing environmental contamination risk. J Hosp Infect.

[31] Johnson D.L., Mead K.R., Lynch R.A., Hirst D.V. (2013). Lifting the lid on toilet plume aerosol: a literature review with suggestions for future research. Am J Infect Control.

[32] Crimaldi J.P., True A.C., Linden K.G., Hernandez M.T., Larson L.T., Pauls A.K. (2022). Commercial toilets emit energetic and rapidly spreading aerosol plumes. Sci Rep.

[33] Heireman L., Hamerlinck H., Vandendriessche S., Boelens J., Coorevits L., De Brabandere E. (2020). Toilet drain water as a potential source of hospital room-to-room transmission of carbapenemase-producing *Klebsiella pneumoniae*. J Hosp Infect.

[34] Andrews V., Hasman H., Midttun M., Feldthaus M.B., Porsbo L.J., Holzknecht B.J. (2021). A hospital outbreak of an NDM-producing ST167 *Escherichia coli* with a possible link to a toilet. J Hosp Infect.

[35] Smismans A., Ho E., Daniels D., Ombelet S., Mellaerts B., Obbels D. (2019). New environmental reservoir of CPE in hospitals. Lancet Infect Dis.

[36] Ikhimiukor O.O., Souza S.S.R., Akintayo I.J., Marcovici M.M., Workman A., Martin I.W. (2023). Phylogenetic lineages and antimicrobial resistance determinants of clinical *Klebsiella oxytoca* spanning local to global scales. Microbiol Spectr.

[37] Long H., Hu Y., Feng Y., Zong Z. (2022). Genome analysis of *Klebsiella oxytoca* complex for antimicrobial resistance and virulence genes. Antimicrob Agents Chemother.

